# Electron Dynamics with Explicit-Time Density Functional Theory of the [4+2] Diels–Alder Reaction

**DOI:** 10.1021/acs.jctc.9b00690

**Published:** 2020-02-24

**Authors:** Angela Acocella, Tainah D. Marforio, Matteo Calvaresi, Andrea Bottoni, Francesco Zerbetto

**Affiliations:** Department of Chemistry “G. Ciamician”, Alma Mater Studiorum − University of Bologna, via Selmi 2, Bologna 40126, Italy

## Abstract

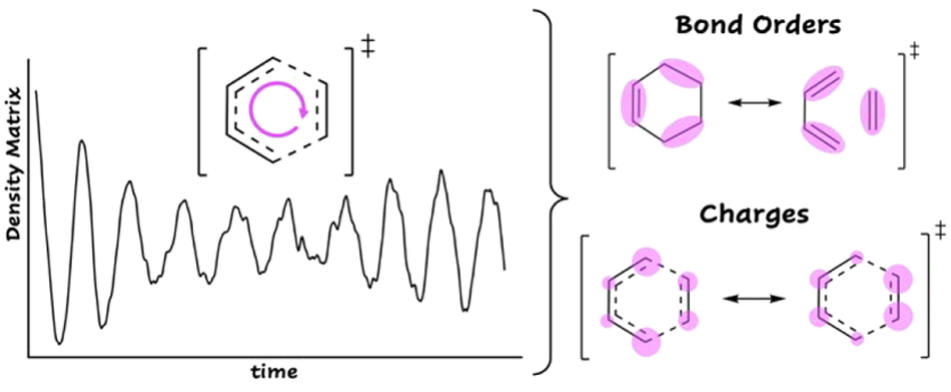

The prototype Diels–Alder (DA) reaction between butadiene and ethene (system **1**) and the DA reaction involving 1-methoxy-butadiene and cyano-ethylene (system **2**) are investigated with an explicit-time-dependent Density Functional Theory approach. Bond orders and atomic net charges obtained in the dynamics at the transition state geometry and along the reaction coordinate toward reactants are used to provide a picture of the process in terms of VB/Lewis resonance structures that contribute to a resonance hybrid. The entire dynamics can be divided into different domains (reactant-like, product-like, and transition state domains) where different Lewis resonance structures contribute with different weights. The relative importance of these three domains varies along the reaction coordinate. In addition to the usual reactant-like and product-like covalent Lewis structures, ionic Lewis structures have non-negligible weights. In system **2**, the electron-donor OCH_3_ on the diene and the electron-acceptor CN on the dienophile make more important the contributions of ionic Lewis structures that stabilize the transition state and determine the decrease of the reaction barrier with respect to system **1**.

## Introduction

Molecules, due to the ever-present electron delocalization, can be represented as resonance hybrids to which Lewis (resonance) structures contribute with different weights.

The hybrid mesomeric wave function Ψ is described as a linear combination of Lewis structures, ϕ_*i*_:
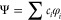
1where *c*_*i*_ are variational coefficients that represent the weight of ϕ_*i*_ and minimize the electronic energy. Because the energy of a resonance hybrid is lower than the energy of any of the individual Lewis structures, much of the chemical stability and reactivity can be rationalized in terms of mesomerism. High energy states, such as transition states (TSs), can be stabilized by the presence of various resonance structures with a consequent lowering of the activation barrier. Therefore, mesomerism helps to explain why some reactions are fast and others are slow or do not proceed at all. “Arrow chemistry” is the pictorial representation of mesomerism. This qualitative popular approach to mesomerism was demonstrated to be a powerful tool capable of rationalizing organic reactivity. Arrows indicate the motion of individual or pairs of electrons connecting different Lewis structures and, in a natural extension, leading to products from reactants.

The contributions *c*_*i*_ of each resonance (Lewis) structure ϕ_*i*_ can be, in principle, computed with the Valence Bond (VB) theory, ascribable to the pioneering work of Ingold and Pauling.^1−4^ The pictorial movement of electrons, described by the curly arrows, can be related to the oscillations of the electronic wave function, which even in the stationary case can be caused by vibrations or by the environment. This electron density reorganization occurs in a subfemtosecond time scale (where nuclei are frozen) and can be related to the contributions of the various resonance structures ϕ_*i*_’s.

The VB approach is seldom used because of the high complexity and computational costs required for VB calculations. Instead, Molecular Orbital (MO) theory is routinely employed to determine Ψ, but it can give only a rough estimate of the contribution of the various resonance (Lewis) structures. In this way, the familiar description of reactivity in terms of Lewis structures and curly arrows (beloved by organic chemists) is almost completely missed. Nevertheless, in a few papers, the language of mesomerism was used to interpret the results of MO computations for some prototype organic reactions.^5−7^ Bernardi and co-workers computed resonance energies defined in the theory of aromaticity using a VB Hamiltonian obtained from CASSCF wave functions. They applied this analysis to the transition structures of ethylene dimerization and Diels–Alder (DA) reaction between ethylene and butadiene.^5^ More recently, the prototype DA reaction was investigated with a new approach able to extract the movement of the electrons from static (no-time-dependent) Hartree–Fock calculations that were used to construct a reference configuration and “excited” configurations.^6^ For the Claisen rearrangement and other reactions, the bond reorganization expressed by curly arrows was directly observed in ab initio calculations as transformations of intrinsic bond orbitals along the reaction coordinate.^7^

Even if it is commonly accepted that there is no time-dependent oscillation between the resonance structures (ϕ_*i*_ partakes in Ψ, but not as a function of time), time is implicitly present in “arrow chemistry”. The arrows indicate how electrons move and reorganize in the reactants–products transformation and are an example of time dependence.

To evaluate the contribution of the Lewis structures to the resonance hybrid and renovate the language of mesomerism in the context of MO computations, a time-dependent picture where electrons reorganize at the ultrafast time scale can be employed. In this Article, we use an explicit-time Density Functional Theory approach to obtain a “mesomeric” picture of one of the most popular pericyclic reactions: the [4+2] Diels–Alder (DA) cycloaddition. We investigate two different systems: (i) the prototype DA reaction between butadiene and ethylene (system **1**), and (ii) the reaction between 1-methoxy-butadiene and cyano-ethylene, labeled as system **2**. The RT-TDDFT (real-time-dependent Density Functional Theory) dynamics, carried out at fixed nuclei, describes the change in time of electron densities in TSs and intermediate points along the Intrinsic Reaction Coordinate (IRC). Within the present theoretical approach, the initial nonstationary wave function, describing the two unperturbed fragments at the TS (or IRC point) geometry, is perturbed by the adiabatic Hamiltonian, which represents the effect of the working/chemical environment upon the molecular fragments. The perturbation activates the electron flow between and within the fragments. During the dynamics, the total density matrix population shifts between diagonal and off-diagonal elements and provides information on the evolution of local atomic charges (*Q*’s) and bond orders (BOs). Because our dynamics does not provide the values of experimental observables, bond orders and atomic charges, which are related to the evolution of the wave function, are useful quantities to discuss many experimental results for the DA reaction and not directly measured by the dynamics. In this picture, BOs and *Q*’s vary in time, they identify the resonance structures ϕ_*i*_’s explored by the hybrid Ψ, and they are crucial to decode the nature of the wave function in terms of VB structures.

## Computational Background

The explicit-time-dependent DFT simulations were performed by means of a numerically stable algorithm that we applied in previous studies to predict the nonlinear electronic response in systems under the effect of external perturbations:^8−16^
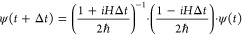
2where ψ(*t*) is the nonstationary wave function at time(*t*), *H* is the Hamiltonian, and Δ*t* is the simulation time-step, here set to 0.0048 fs. The time dependence of the electronic wave function is calculated using a generalized Cayley algorithm,^17^ based on a Dyson-like expansion of the time-evolution operator,^18^ which conserves probability and preserves orthogonality. Numerically, the algorithm evolves the time-dependent Schrödinger equation by means of the Crank–Nicolson method.^19,20^

Wave function coefficients and energies of the critical and intermediate geometries computed on the ground-state PES of systems **1** and **2**, as reported in the QM section of the Supporting Information, were used to set up the wavepacket dynamics, in a vacuum. Initially, the wave function, ψ(*t* = 0), is built in terms of block-localized MOs for each fragment, that is, the diene (DN) and dienophile (DP). The ground-state electronic structures of both fragments, distinctly obtained on their frozen geometries at different PES points, were calculated at the M06-2X/6-31+G(d) level of theory:
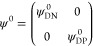
3

In a sense, the initial wave function is constrained on the electronic structures of the unperturbed fragments along the nuclear geometries of the reaction path. The ψ_DN_^0^ and ψ_DP_^0^ wave functions were orthonormalized by a Löwdin transformation.

The coupling between fragments is introduced by means of the electronic Hamiltonian calculated at the same level of theory on the Löwdin orthonormalized wave functions of the total system at each PES geometry.

To quantify the electron density redistribution activated by coupling between interacting fragments, bond orders and atomic effective charges were calculated in time for different PES points.

In particular, the time-dependent inter- and intramolecular bond order between atoms A and B was calculated from the total density matrix *P*(*t*), according to the Wiberg definition:^21−23^
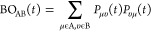
4

The time-dependent atomic effective charge on atom A, for an atom centered basis set, was estimated by the Lödwin population analysis:
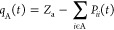
5

The orbital occupation numbers, determined by projecting the time-dependent density matrix onto the initial orbitals, were also calculated:
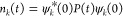
6

We consider short-time coherent electron dynamics, of about 5 fs time length, before vibrational relaxation and interactions with the environment occur.

Importantly, the wave function ψ(*t* + Δ*t*) of eq 2 is nonvariational. If ψ(*t* = 0) is the eigenfunction of *H*, the dynamics is trivial and does not require numerical integration. In the present context, either the Hamiltonian or the wave function can be adiabatic:
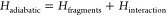
7
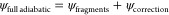
8where
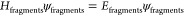
9

However, *H*_interaction_ and ψ_correction_ are not related by an eigenvalue problem. In a perturbative scheme, ψ_correction_ can include contributions from electronic excitations from the ψ(*t* = 0) wave function. These excitations effectively make ψ(*t*) multiconfigurational. Analogously, *H*_interaction_ includes perturbative contributions at all orders of perturbation.

In the present calculations, *H*_adiabatic_ operates on ψ_fragments_.

## Results and Discussion

### QM Calculations

**Figure 1:**
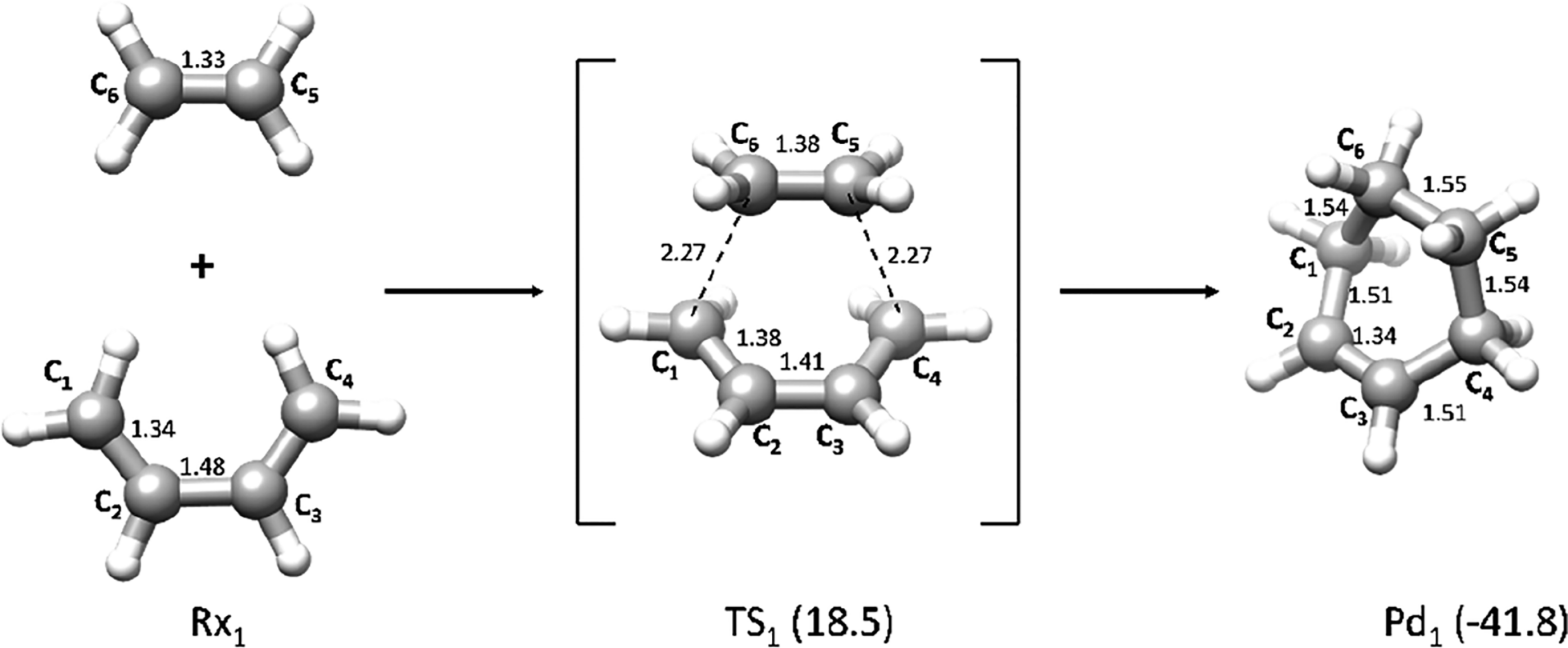
Prototype Diels–Alder reaction between butadiene and ethylene. Energies (kcal mol^–1^) relative to reactants Rx_1_ are reported in parentheses and include zero-point corrections. Bond lengths are in angströms.

**Figure 2:**
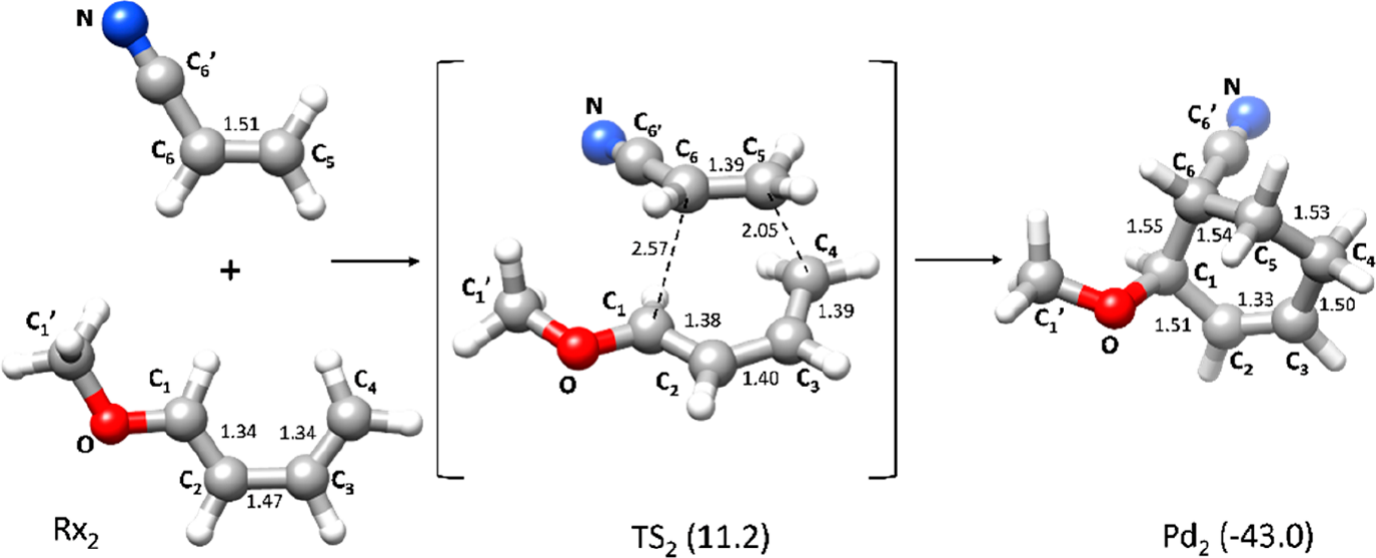
Diels–Alder reaction between 1-methoxy-butadiene and cyano-ethylene leading to 3-methoxy-4-cyano-cyclohexene. Energies (kcal mol^–1^) relative to reactants Rx_2_ are reported in parentheses and include zero-point corrections. Bond lengths are in angströms.

All calculations were performed in vacuo with the M06-2X functional^24^ with the 6-31+G(d) basis set. A schematic representation of the critical points obtained for system **1** and system **2** is reported in Figures 1 and 2.

Following the usual interpretation of a [4π+2σ] pericyclic reaction, the prototype DA reaction between butadiene and ethylene (system **1**) is a concerted process (as confirmed by previous studies^25^). The transition state (TS_1_) is a cyclic symmetric aromatic-like structure, where two new σ C–C bonds between C_1_(C_4_) and C_6_(C_5_) (2.27 Å) form simultaneously to the elongation of the C_1_–C_2_(C_3_–C_4_) π-bonds (1.38 Å). The C_2_–C_3_ bond shortens from 1.47 Å in Rx_1_ to 1.41 Å in TS_1_. The energy barrier from Rx_1_ is 18.5 kcal mol^–1^. The formation of product Pd_1_ is highly exothermic (−41.8 kcal mol^–1^).

The DA reaction mechanism computed for the formation of 3-methoxy-4-cyano-cyclohexene from 1-methoxy-butadiene and cyano-ethylene (system **2**) entails the passage through transition state TS_2_ where the new C–C bonds form asymmetrically. TS_2_ corresponds to the most favorable relative orientation of the two reactant molecules. All other possible orientations were discarded because they require a significantly higher activation energy (see Table S1). The σ-bond C_4_–C_5_ (2.05 Å) is almost formed when C_1_ and C_6_ are rather distant (2.57 Å). The energy of TS_2_ with respect to reactants Rx_2_ is 11.2 kcal mol^–1^, and the exothermicity of the reaction is 43.0 kcal mol^–1^.

### Wave Function Dynamics

As previously underlined, the results of our theoretical approach cannot be directly compared to the experiment. Thus, to interpret in terms of VB/Lewis structures the experimental evidence available for DA reactions and the results of MO computations, we used bond orders (BOs) and atomic net charges (*Q*’s) obtained during the dynamics. In system **1**, bond orders, BOs, and charges, *Q*’s, are weakly periodic, as shown in Figure S1 and Scheme S1. The period of their oscillations is less than a femtosecond. In the following discussion, we refer to the BOs and *Q*’s of the reactants (products) and transition state reported in Table 1 and in Tables S2–S5. The values for Rx_1_ (Pd_1_) were obtained from the DFT static computations, while the results reported for TS_1_ refer to 5 fs of dynamics carried out at the transition state geometry. The butadiene double bonds C_1_–C_2_ and C_3_–C_4_ have a BO value of 2.11 (1.15) in Rx_1_ (Pd_1_). During the dynamics, it oscillates between 2.03 and 1.31, with an average value of 1.60. The butadiene single bond C_2_–C_3_ has a BO value of 1.23 (2.04) and oscillates between 1.80 and 1.14 (with an average value of 1.42) in the simulation. The ethene bond C_5_–C_6_ has a BO value of 2.30 (1.15) in reactants (products) and oscillates between 2.26 and 1.21, with an average value of 1.65. The incipient bond has a BO value of 0.0 (1.13) and oscillates between 0.0 and 0.83 during the dynamics, with an average value of 0.42. These values are collected in Table S2.

**Table 1:** Percentages of the Dynamics Computed for Different Ranges of BOs at the Transition State TS_1_ and for Various Points, IP_*n*_, Determined along the IRC in the Reactant Direction

domain	bond	BO range	TS_1_	IP_1_	IP_2_	IP_3_	IP_4_	IP_5_
reactant-like	C_4_C_5_(C_1_C_6_)	≤0.25	8%	22%	45%	75%	93%	99%
	C_2_C_3_	≤1.25						
trans. state/benzene-like	C_4_C_5_(C_1_C_6_)	[≥0.25, ≤0.55]	72%	72%	55%	25%	7%	1%
	C_2_C_3_	[≥1.25, ≤1.55]						
product-like	C_4_C_5_(C_1_C_6_)	≥0.55	20%	6%	0%	0%	0%	0%
	C_2_C_3_	≥1.55						

If we choose the average value as a reference and consider a deviation of ±20% of the difference between the maximum and minimum values of each BO during the dynamics (see Table S2), we find that for 72% of the dynamics the C_2_–C_3_ BO ranges from 1.25 to 1.55 and the incipient C_4_–C_5_(C_1_–C_6_) BO is in the range from 0.25 to 0.55. These results are collected in Table 1.

**Scheme 1:**
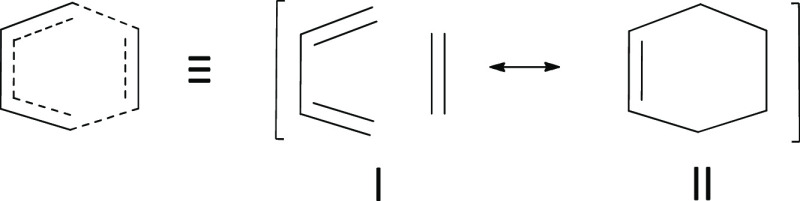
A Schematic Representation of the Two Covalent Lewis Structures for the Prototype DA Reaction

Thus, assuming the BOs of C_2_–C_3_ and C_4_–C_5_(C_1_–C_6_) as meaningful indicators of the time evolution of electron motion, the electron distribution observed in the major part of the dynamics (72%) is represented by a resonance hybrid where the inner butadiene bond is becoming single and the incipient bond is in an advanced state of formation. This hybrid is a linear combination of various Lewis structures that must include covalent structures **I** and **II** of Scheme 1, with significant and comparable weights.

The limiting values of the oscillations reach close to the values of the reactants and product, suggesting a different contribution of **I** and **II** in various intervals of the dynamics. When in the oscillations, a large value of the BO of the incipient bond is associated with a large value of the inner C_2_–C_3_ butadiene bond (this occurs for 20% of dynamics, with BOs of C_4_–C_5_(C_1_–C_6_) > 0.50 and C_2_–C_3_ > 1.50), the Lewis structure **II** is dominant, and the corresponding resonance hybrid is product-like.

**Figure 3:**
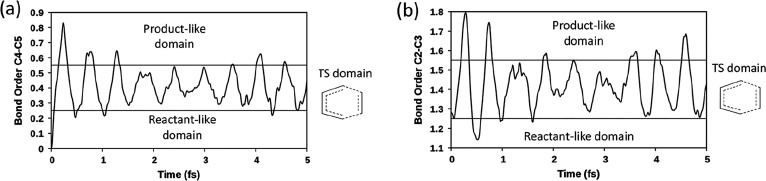
Time-dependency of (a) C_4_–C_5_(C_1_–C_6_) BO and (b) C_2_–C_3_ BO. The transition state (benzene-like) domain corresponds to the central horizontal zone.

The contribution of **I** and **II** is reversed (**I** becomes dominant) when the BOs of C_2_–C_3_ and incipient bonds significantly decrease (BO of C_2_–C_3_ ≤ 1.25 and BO of C_4_–C_5_(C_1_–C_6_) ≤ 0.25). This occurs for 8% of dynamics, and the resonance hybrid becomes reactant-like. Thus, the entire dynamics can be thought of as divided into different domains: reactant-like, product-like, and transition state domains. We can name the central domain as a benzene-like domain because of the complete electron delocalization. A graphical representation of these three domains is given in Figure 3, reporting the time-dependency of C_4_–C_5_(C_1_–C_6_) and C_2_–C_3_ BOs (left and right sides of the diagram, respectively). The transition state domain corresponds to the central horizontal region of the diagrams, where the C_4_–C_5_(C_1_–C_6_) BO ranges from 0.25 to 0.55 and the C_2_–C_3_ BO from 1.25 to 1.55. The upper and lower regions represent the product-like and reactant-like domains. The transition state domain is marked by a benzene-like structure showing the concerted, synchronous character of the mechanism.

The 5 fs dynamics were carried out for various points along the Intrinsic Reaction Coordinate (IRC) in the reactant direction (see Table 1). Energy values along the IRC are reported in Table S3. Importantly, as the system approaches the reactants, the percentage of dynamics characterized by a BO of the incipient bond C_4_–C_5_(C_1_–C_6_) ≤ 0.25 and of the inner C_2_–C_3_ bond ≤ 1.25 increases significantly (from 8% to 99%); that is, the reactant-like domain becomes rapidly more important. Simultaneously, we observed a decrease of the percentage corresponding to C_4_–C_5_(C_1_–C_6_) BO ≥ 0.25 and C_2_–C_3_ BO ≥ 1.25. This trend perfectly agrees with the gradual increase of the weight of Lewis structure **I** and the concurrent decrease of the importance of structure **II**.

**Scheme 2:**
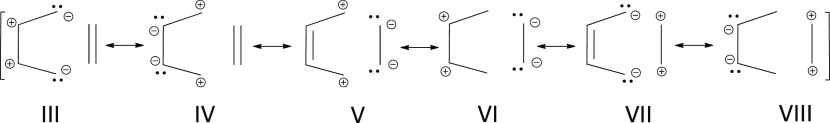
A Schematic Representation of the Possible Ionic Lewis Structures for the Prototype DA Reaction In a molecular orbital approach, some structures are multiconfigurational.

The analysis of the atomic charges computed in the dynamics (see Table S4) can identify qualitatively the contribution of other Lewis resonance structures bearing formal negative and positive *Q*’s on the atoms (see Scheme 2).

The terminal carbon atom of butadiene is characterized by a negative charge that is on average −0.47, more negative than the charge in reactants (products), which is −0.42 (−0.41). The increase of negative charge with respect to reactants (the maximum charge on C_1_(C_4_) is −0.32, while the minimum value is −0.68) suggests that almost one electron occasionally becomes localized on these atoms and indicates that ionic Lewis structures such as **III** and **VII** provide a non-negligible contribution to the resonance hybrid describing the transition state.

The charge of the inner carbon has an average value of −0.28, which is similar to that in reactants (products) that is −0.27 (−0.28). It ranges between −0.03 and −0.51, consistent with contributions of structures **IV**, **V**, **VII**, and **VIII**. The charge of the ethene carbons C_5_(C_6_) is on average −0.42, similar to that of the reactants (products) that is −0.44 (−0.42). In the simulation, this charge varies between −0.16 and −0.67, which is compatible with contributions of structures **V** and **VI**.

Thus, during the simulation, both C_1_(C_4_) and C_5_(C_6_) become either more positive or more negative with respect to reactants, suggesting that butadiene and ethylene can behave either as a donor (nucleophile) or as an acceptor (electrophile).

**Figure 4:**
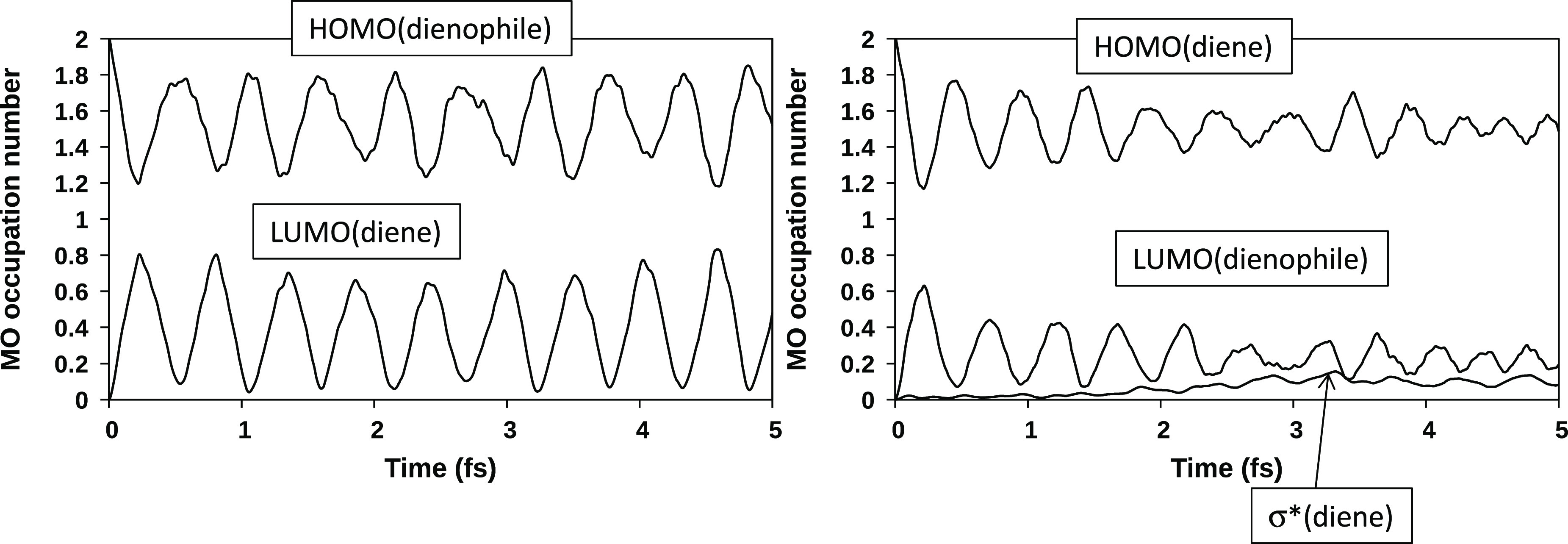
Electronic occupancies at the TS_1_ geometry over the 5 fs dynamics of the dienophile HOMO and diene LUMO and of the diene HOMO (left side), and of the dienophile LUMO and the C_2_–C_3_σ* orbital (right side).

However, the increase of positive charge on C_5_(C_6_) is larger with respect to C_1_(C_4_) (−0.16 and −0.32 are the less negative values, respectively, and −0.42 and −0.47 are the corresponding average charges). Thus, the simulation shows that electrons have a stronger propensity to move from ethene to butadiene than in the opposite direction, suggesting that, at the transition state geometry, ethene is playing a more important role as a donor than butadiene. The trend of occupancies of the frontier orbitals can help to understand the difference between diene and dienophile. During 5 fs of dynamics (see Figure 4), the occupancies of the dienophile HOMO and diene LUMO show a regular and opposite oscillating trend in the ranges 0.8–0.2 and 1.2–1.8, respectively.

The sum of the two occupancies is approximately 2. Thus, the two orbitals “are talking directly to each other”, without involvement of other orbitals. A different behavior was observed for the occupancies of the diene HOMO and dienophile LUMO. During the first 2 fs of dynamics, the trend is similar to that previously discussed. After this time interval, the curves become flatter, and the involvement in the charge transfer of the σ* orbital of the inner (C_2_–C_3_) butadiene bond is evident. Thus, electrons are also moving within the butadiene fragment from the HOMO to the σ* orbital. These orbitals, at the transition state geometry, have the correct symmetry to interact because they are both antisymmetric with respect to the symmetry plane that characterizes the TS_1_ structure. The effect of this intrafragment charge transfer is that during the simulation butadiene appears as a worse donor than ethylene.

Additional information on the nature of the transition state hybrid can be obtained from the percentages of the dynamics corresponding to different ranges of *Q*’s at the transition state TS_1_ and for various points along the IRC. These data are reported in Table S5 and discussed in detail in the Supporting Information. They demonstrate that structures such as **V**, **VI**, and **VII** must be taken into account to provide an accurate description of the resonance hybrid corresponding to the transition state.

Bond orders for reactants (product) and transition state for system **2** are collected in Table S6. Also, for system **2**, bond orders and charges are weakly periodic (Figure S2 and Scheme S2). The BO of the stronger incipient bond C_4_–C_5_ oscillates between 0.0 and 0.90. Its average value, 0.55, is greater than that (0.42) calculated for the symmetric pathway of system **1**. For the weaker incipient bond C_1_–C_6_, BO oscillates in the range 0.0–0.39, with an average value of 0.20, much lower with respect to that of system **1**, reflecting the asynchronous character of TS_2_.

**Scheme 3:**
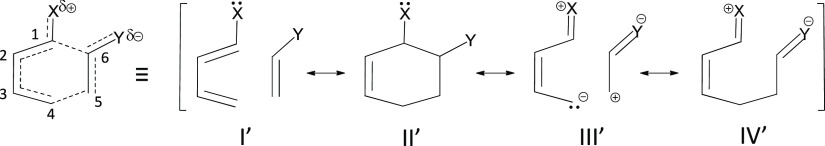
A Schematic Representation of Relevant Lewis Structures for the DA Reaction Occurring in System **2** X = OCH_3_, Y = CN.

The butadiene double bond C_1_–C_2_ in reactants has a BO of 1.89, lower with respect to system **1** (2.11). This is consistent with the expected effect of the electron-donating group OCH_3_ and the consequent contribution of Lewis structures such as **III′** in Scheme 3.

The effect of this group and the influence of structure **III′** on the transition state resonance hybrid are evident during the dynamics because the C_1_–C_2_ BO varies in the range 1.74–1.22 with an average value of 1.47 (it was 1.60 in system **1**). The BO value of the butadiene single bond C_2_–C_3_ in reactants (products) is 1.21 (2.01) and during the dynamics oscillates between 1.71 and 1.18, with an average value of 1.44, larger than that found in system **1** (1.42). Even in this case, the influence of structure **III′** is evident. Finally, the ethene bond C_5_–C_6_ has a BO value of 2.10 (1.09) in reactants (products) and during the dynamics oscillates between 2.05 and 1.18 (average value of 1.49). The decrease of bond order with respect to system **1** (average value of 1.65) is coherent with the presence of the electron-withdrawing group CN and a non-negligible contribution of **III′** and **IV′** (Scheme 3).

In Table 2, we report the percentages of the dynamics corresponding to different ranges of the BOs for C_2_–C_3_ and C_4_–C_5_. Deviations of ±20% of the difference between maximum and minimum were used again to define the different domains.

**Table 2:** Percentages of the Dynamics Computed for Different Ranges of BOs at the Transition State TS_2_ and for Various Points IP_*n*_ Determined along the IRC toward the Reactants

domain	bond	BO range	TS_2_	IP1	IP_2_	IP_3_	IP_4_	IP_5_
reactant-like	C_4_C_5_	≤0.40	20%	29%	45%	61%	99%	100%
	C_2_C_3_	≤1.25						
trans. state/benzene-like	C_4_C_5_	≥0.40, ≤0.70	49%	54%	51%	39%	4%	0%
	C_2_C_3_	≥ 1.25, ≤1.55						
product-like	C_4_C_5_	≥0.70	31%	17%	4%	0%	0%	0%
	C_2_C_3_	≥1.55						

For 49%, the C_2_–C_3_ BO and the C_4_–C_5_ BO vary in the intervals 1.25–1.55 and 0.40–0.70, respectively. Thus, for a significant percentage of the dynamics, electrons are “exploring” a resonance hybrid where the contributions of Lewis structures of Scheme 3 (in particular, **II′**, **III′**, and **IV′**) are significant. Also, the contribution of structure **IV′** (a five-centers product-like structure) is consistent with the fact that the incipient C_1_–C_6_ bond is nearly absent in the transition state resonance hybrid. Importantly, Lewis structures such as **III′** and **IV′** are not present in system **1**. In system **2**, these structures contribute to stabilize further the transition state and lower the activation barrier. For 20% of dynamics, the C_2_–C_3_ BO is smaller than 1.25 and the C_4_–C_5_ BO is smaller than 0.40. This domain corresponds to a reactant-like hybrid structure, where Lewis structures such as **I′** and **III′** are dominant. 31% of dynamics is spent by electrons exploring a product-like resonance hybrid where the major contribution is represented by **II′**. The time-dependency of C_2_–C_3_ and C_4_–C_5_ BOs used as indicators of the time evolution of the electron flow is shown in Figure S3. Transition state, reactant-like, and product-like domains are evidenced.

The results of dynamics carried out along the IRC toward the reactants (Table 2) are again informative. In approaching the reactants, the time spent by electrons in exploring a reactant-like hybrid structure (C_2_–C_3_ BO < 0.40 and C_4_–C_5_ BO < 1.25) rapidly increases: there is a larger contribution of **I′** and **III′** to the hybrid resonance with a simultaneous decrease of Lewis structures **II′** and **IV′**.

**Scheme 4:**
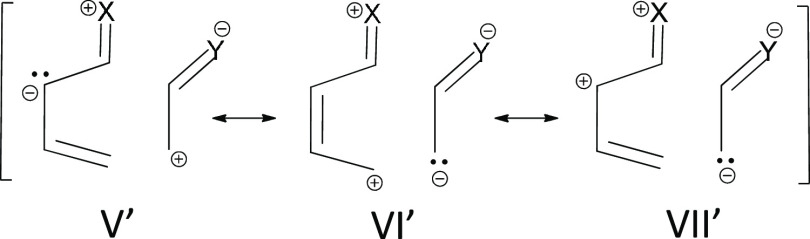
Schematic Representation of Ionic Lewis Structures for the DA Reaction Occurring in System **2** X = OCH_3_, Y = CN.

A more complete picture appears when local charges are considered (see Table S7). The terminal carbon atom of butadiene (C_4_) that forms the stronger incipient bond has a negative charge that is on average −0.44 (it oscillates between −0.24 and −0.66), identical to the charge in reactants. The terminal carbon atom of butadiene that forms the weaker incipient bond (C_1_) is often nearly neutral (it oscillates between 0.08 and −0.29), its average charge being −0.09. The charges of the two atoms oscillate out-of-phase (see Figure S3, right panel). The charge on the ethene carbon C_5_, involved in the stronger new bond, is on average −0.41 (it oscillates between −0.23 and −0.56), more negative with respect to reactants (−0.34). Because during the simulation both C_4_ and C_5_ can become either more positive or more negative with respect to reactants and the atomic charges oscillate out-of-phase, these two atoms can behave either as a nucleophilic or as an electrophilic center. All of the above evidence enforces the idea that Lewis structures such as **III′** and **IV′** strongly participate in the resonance hybrid that represents the transition state, but additional contributions, even though less important, arise from Lewis structures such as **V′**, **VI′**, and **VII′** (Scheme 4). However, because the increase of negative charge with respect to reactants is larger on C_5_ (ethene) than on C_4_ (butadiene) (previously reported average charges are −0.41 and −0.44, respectively), the dynamics indicates that, at the transition state geometry, electrons spend more time on ethene than on butadiene. In other words, and contrary to what was found for system **1**, butadiene becomes a better donor than ethene, in agreement with the presence on butadiene of the electron-donating group OCH_3_.

**Figure 5:**
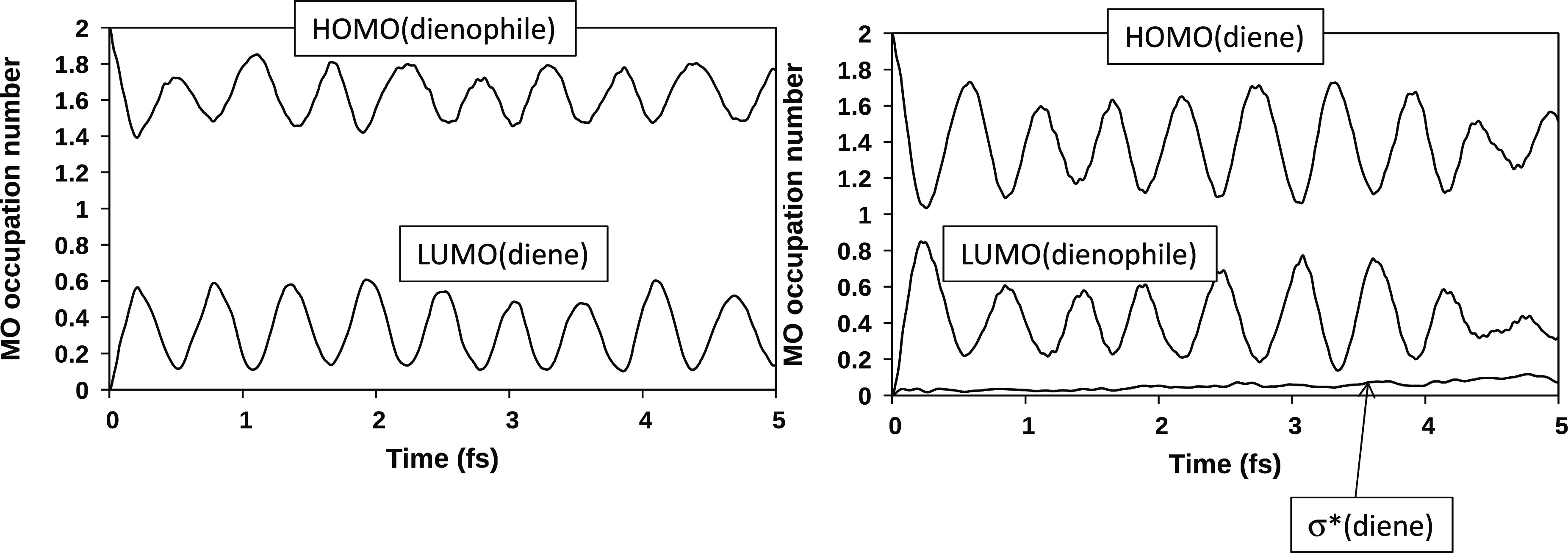
Electronic occupancies at the TS_2_ geometry over the 5 fs dynamics of the dienophile HOMO and diene LUMO (left side) and of the diene HOMO, the dienophile LUMO, and the C_2_–C_3_σ* orbital (right side).

Further evidence of the effect of substituents on diene (OCH_3_) and dienophile (CN) is provided by the trend of occupancies of the frontier orbitals during the dynamics (Figure 5) and comparison with system **1**. In system **1**, the oscillating trends of the dienophile HOMO and diene HOMO are similar: at the beginning of the dynamics, the corresponding occupancies vary from 2 to ∼1.2, which indicates the transfer of ∼0.8 of an electron. In system **2**, these two orbitals show a rather different behavior: the occupancy of the diene HOMO (right side of Figure 5) changes from 2 to 1.1, while that of the dienophile HOMO (left side) changes from 2 to 1.4. Thus, the charge transfer originating from the dienophile is significantly lower than that coming from the diene, and this in turn is slightly higher with respect to system **1**. This trend is consistent with the presence of the electron-donating group OCH_3_ on the diene and the electron-withdrawing group CN on the dienophile, which partly “adsorbs” the moving electrons.

Even if the results of our dynamics are not directly connected to the experiment, we have shown that bond orders and charges can be used to interpret experimental evidence in terms of VB structures. As a further example, we use the VB picture derived by BOs and *Q*’s to elucidate some interesting experimental results obtained by Bartlett^26^ at the end of the 1960s and concerning the mechanism (concerted or two-step) of the DA reaction. This author demonstrated that in the case of few alkenes (for instance, fluorinated alkenes) the reaction with butadiene leads exclusively to four-membered rings (1,2 cycloaddition). In other cases, a mixing of four-membered and six-membered (1,4-cycloaddition) rings was observed. Furthermore, a careful reinvestigation of the prototype DA reaction (ethylene + butadiene) revealed, besides the dominant cyclohexene product, 0.02% of vinylcyclobutane. Also, the reaction is usually stereospecific, suggesting a concerted mechanism. However, such stereospecificity often disappears in the formation of four-membered adducts. These results are consistent with the existence of two possible reaction paths, a concerted path and a nonconcerted one involving a biradical or dipolar ion intermediate. The lifetime of this intermediate can be long enough to allow the internal rotation to compete with ring closure with loss of stereospecificity. The existence of a potentially competitive nonconcerted path involving a dipolar ion and leading to four-membered adducts is evidenced by VB structures **VI** and **VIII** in Scheme 2 and structures **V′** and **VII′** in Scheme 4. These structures are usually high in energy (for instance, in the prototype DA reaction). They can stabilize in the presence of appropriate electron-withdrawing and electron-donating substituents, making the 1,2 cycloaddition path more favorable. In some cases, the two reaction paths coexist (mix of 1,4 and 1,2 adducts), while in other cases (for instance, fluorinated ethylenes) the 1,2 reaction channel can become dominant.

## Conclusions

Explicit-time-dependent Density Functional Theory was used to investigate the prototype [4+2] DA reaction between butadiene and ethylene (system **1**) and the DA reaction between 1-methoxy-butadiene and cyano-ethylene (system **2**). Analysis of bond orders (BOs) and atomic net charges (*Q*’s) during the dynamics allows one to interpret the results of MO computations in terms of VB/Lewis structures.

The entire dynamics obtained for system **1** can be divided into three domains: reactant-like, product-like, and transition state domains. The transition state domain is characterized by a complete electron delocalization (benzene-like domain). It corresponds to a resonance hybrid that can be represented as a linear combination of various Lewis structures. Covalent reactant-like and product-like structures of Scheme 1 are dominant and contribute with comparable weights. Besides conventional covalent Lewis structures, other (ionic) Lewis structures (Scheme 2) give non-negligible contributions.

The relative importance of all VB structures varies along the reaction coordinate. The transition state domain is dominant at the transition state geometry, but its importance rapidly decreases when we move from transition state to reactant geometry. Simultaneously, the importance of the reactant-like domain (a resonance hybrid dominated by Lewis covalent structure **II**) increases.

In system **2**, additional Lewis structures involving the electron-donor OCH_3_ on diene and the electron-acceptor CN on dienophile give important stabilizing contributions to the transition state resonance hybrid (Scheme 3) and determine a decrease of the reaction barrier with respect to the prototype case.

Our results indicate that during the dynamics both diene and dienophile can behave either as an electron donor or as an electron acceptor. For the prototype reaction, because of an internal electron transfer mechanism involving the diene HOMO and the σ* orbital of the inner (C_2_–C_3_) diene bond, ethylene appears as a better donor than butadiene. The situation is reversed in system **2** because of the presence of the two substituents on diene (OCH_3_) and dienophile (CN).

The VB picture derived by BOs and *Q*’s helps to elucidate some unusual experimental results^26^ showing that four-membered rings can be obtained in the presence of specific substituents (F, Cl, CN) on the dienophile. VB structures such as **VI** and **VIII** in Scheme 2 (system **1**) and **V′** and **VII′** in Scheme 4 (system **2**) are consistent with the existence of a competitive nonconcerted path involving a dipolar ion intermediate and leading to four-membered adducts with loss of stereospecificity.

The result presented here are rather promising for possible future applications of explicit-time Density Functional Theory to chemical reactivity and its interpretation in terms of VB language.

## Supporting Information Available

Computational details for QM calculations and RT-TDDFT wavepacket dynamics; activation energies computed for eight different approaches of 1-methoxy-butadiene and cyano-ethylene; maximum, minimum, and average bond orders and Löwdin charges, over 5 fs of dynamics, for systems **1** and **2**; and Cartesian coordinates for reactants, products, transition states, and IRC points for systems **1** and **2** (PDF)

## References

[ref1] Ingold C. K. Principles of an electronic theory of organic reactions. Chem. Rev. 1934, 15, 225–274. 10.1021/cr60051a003.

[ref2] Pauling L.; Wheland G. W. Nature of the chemical Bond V. The quantum-mechanical calculation of resonance energy of benzene and naphthalene and the hydrocarbon free radicals. J. Chem. Phys. 1933, 1, 362–374. 10.1063/1.1749304.

[ref3] Pauling L.; Sherman J. Nature of the chemical Bond VI. The calculation from thermochemical data of the energy of resonance of molecules among several electronic structures. J. Chem. Phys. 1933, 1, 606–617. 10.1063/1.1749335.

[ref4] Pauling L.; Sherman J. Nature of the chemical Bond VII. The calculation of resonance energy on conjugated systems. J. Chem. Phys. 1933, 1, 679–686. 10.1063/1.1749226.

[ref5] Bernardi F.; Celani P.; Olivucci M.; Robb M. A.; Suzzi-Valli G. Theoretical study of the Aromatic character of the transistion-state of allowed and forbidden cycloadditions. J. Am. Chem. Soc. 1995, 117, 10531–10536. 10.1021/ja00147a014.

[ref6] Liu Y.; Kilby P.; Frankcombe T. J.; Schmidt T. W. Calculating curly arrows from ab initio wavefunctions. Nat. Commun. 2018, 9, 1–7. 10.1038/s41467-018-03860-2. 29651029 PMC5897577

[ref7] Knizia G.; Klein J. E. M. N. Electron Flow in Reaction Mechanisms Revealed from First Principles. Angew. Chem., Int. Ed. 2015, 54, 5518–5522. 10.1002/anie.201410637. 25737294

[ref8] Acocella A.; Jones G. A.; Zerbetto F. Excitation energy transfer and low-efficiency photolytic splitting of water ice by vacuum UV light. J. Phys. Chem. Lett. 2012, 3, 3610–3615. 10.1021/jz301640h. 26290996

[ref9] Acocella A.; Carbone F.; Zerbetto F. Quantum study of laser-induced initial activation ofgraphite-to-diamond conversion. J. Am. Chem. Soc. 2010, 132, 12166–12167. 10.1021/ja102497z. 20715816

[ref10] Acocella A.; Jones G. A.; Zerbetto F. What is adenine doing in photolyase?. J. Phys. Chem. B 2010, 114, 4101–4106. 10.1021/jp101093z. 20184295

[ref11] Jones G. A.; Acocella A.; Zerbetto F. On-the-Fly, Electric-Field-Driven, Coupled Electron - Nuclear Dynamics. J. Phys. Chem. A 2008, 112 (40), 9650–9656. 10.1021/jp805360v. 18767783

[ref12] Jones G. A.; Acocella A.; Zerbetto F. Nonlinear optical properties of C60 with explicit time-dependent electron dynamics. Theor. Chem. Acc. 2007, 118, 99–106. 10.1007/s00214-007-0251-4.

[ref13] Acocella A.; Jones G. A.; Zerbetto F. Mono- And bichromatic electron dynamics: LiH, a test case. J. Phys. Chem. A 2006, 110, 5164–5172. 10.1021/jp060195i. 16610840

[ref14] Acocella A.; de Simone M.; Evangelista F.; Coreno M.; Rudolf P.; Zerbetto F. Time-dependent quantum simulation of coronene photoemission spectra. Phys. Chem. Chem. Phys. 2016, 18, 13604–13615. 10.1039/C5CP06455D. 27141554

[ref15] Baldini E.; Mann A.; Benfatto L.; Cappelluti E.; Acocella A.; Silkin V. M.; Eremeev S. V.; Kuzmenko A. B.; Tan T.; Xi X. X.; Zerbetto F.; Merlin R.; Carbone F. Real-Time Observation of Phonon-Mediated σ-π Interband Scattering in MgB2. Phys. Rev. Lett. 2017, 119, 1–6. 10.1103/PhysRevLett.119.097002. 28949564

[ref16] Acocella A.; Hofinger S.; Haunschmid E.; Pop S. C.; Narumi T.; Yasuoka K.; Yasui M.; Zerbetto F. Structural determinants in the bulk heterojunction. Phys. Chem. Chem. Phys. 2018, 20, 5708–5720. 10.1039/C7CP08435H. 29410990

[ref17] Allen R. E. Electron-ion dynamics: A technique for simulating both electronic transitions and ionic motion in molecules and materials. Phys. Rev. B: Condens. Matter Mater. Phys. 1994, 50, 18629–18632. 10.1103/PhysRevB.50.18629. 9976300

[ref18] Dumitrica T.; Burzo A.; Dou Y.; Allen R. E. Response of Si and InSb to ultrafast laser pulses. Phys. Status Solidi B 2004, 241, 2331–2342. 10.1002/pssb.200404934.

[ref19] Crank J.; Nicolson P. A pratical method for numerical evaluation of solutions of partial differential equations of the heat-conduction type. Math. Proc. Cambridge Philos. Soc. 1947, 43 (1), 50–67. 10.1017/S0305004100023197.

[ref20] Lorin E.; Bandrauk A. D. Multiresolution scheme for Time-Dependent Schrödinger Equation. Comput. Phys. Commun. 2010, 181, 626–638. 10.1016/j.cpc.2009.11.012.

[ref21] Bridgeman A. J.; Cavigliasso G.; Ireland L. R.; Rothery J. The Mayer bond order as a tool in inorganic chemistry. J. Chem. Soc. Dalt. Trans. 2011, 2095–2108. 10.1039/b102094n.

[ref22] Kalinowski J. A.; Lesyng B.; Thompson J. D.; Cramer C. J.; Truhlar D. G. Class IV Charge model for the self-consistent charge density functional tight-binding method. J. Phys. Chem. A 2004, 108, 2545–2549. 10.1021/jp037288+.

[ref23] Bochiccio R. C.; Reale H. F. On the nature of crystalline bonding: extension of statistical population analysis to two- and three- dimensional crystalline systems. J. Phys. B: At., Mol. Opt. Phys. 1993, 26, 4871–4883. 10.1088/0953-4075/26/24/018.

[ref24] Zhao Y.; Truhlar D. G. The M06 Suite of Density Functionals for Main Group Thermochemistry, Thermochemical Kinetics, Noncovalent Interactions, Excited States, and Transition Elements: Two New Functionals and Systematic Testing of Four M06-Class Functionals and 12 Other Functionals. Theor. Chem. Acc. 2008, 120, 215–241. 10.1007/s00214-007-0310-x.

[ref25] Diau E.; De Feyter S.; Zewail A. H. Femtosecond dynamics of retro Diels-Alder reactions: the concept of concertedness. Chem. Phys. Lett. 1999, 304, 134–144. 10.1016/S0009-2614(99)00315-2.

[ref26] Bartlett P. D. 1,2 and 1,4-Cycloaddition to Conjugated Dienes. Science 1968, 159, 833–838. 10.1126/science.159.3817.833. 17768965

