# Supplementary material for: Electron Dynamics with Explicit-Time Density Functional Theory of the [4+2] Diels–Alder Reaction
Source: J Chem Theory Comput. 2020 Feb 24;16(4):2172–80. doi: 10.1021/acs.jctc.9b00690 (PMC7997368; doi:10.1021/acs.jctc.9b00690)
Supplement: Supplementary file 1 — ct9b00690_si_001.pdf [file ct9b00690_si_001.pdf]

Supporting Information for the paper

Electron dynamics with explicit-time density functional theory of the [4+2] Diels-Alder reaction

Angela Acocella,* † Tainah D. Marforio,* † Matteo Calvaresi, † Andrea Bottoni, † and Francesco Zerbetto†

† Department of Chemistry "G. Ciamician", Alma Mater Studiorum – University of Bologna, via Selmi 2, 40126 Bologna, Italy

Table of Contents:

Computational details
Table S1
Table S2
Table S3
Table S4
Table S5
Comments to Table S5
Table S6
Table S7
Table S8
Comments to Table S8
Table S9
Table S10
Scheme S1
Figure S1
Scheme S2

Figure S2

Figure S3

Computational details

QM calculations. Geometry optimizations were carried out using the M06-2X hybrid meta-GGA density functional[1] as implemented in Gaussian09, Revision D.01[2] on systems **1** and **2**, described in the main text. The performance of DFT in computing geometry, electronic and energetic features of the Diels-Alder reaction has been largely reported in previous studies.[3,4] Houk and co-workers have shown that it is possible to obtain reliable activation energies for pericyclic reactions employing the M06-2X functional.[5,6] The basis set for all computations is a double-ζ (6-31+G(d)).

Structures of the critical points (minima and saddle points) were fully optimized using the gradient method available in the Gaussian package.[2] Frequency calculations were carried out at the same level of theory to verify the nature of the critical points. All transition states (saddle points) were characterized by the presence of one imaginary frequency. IRC computations were also carried out to establish the structure and the energetics of intermediate points along the energy path.

Quantum dynamics. RT-TDDFT wavepacket dynamics

Wavefunction coefficients and energies of the critical and intermediate geometries computed on the ground-state singlet concerted PES of systems **1** and **2**, as reported in the QM section, were used to set-up the wavepacket dynamics, in vacuum. Initially the diabatic wavefunction, $\psi(t=0)$, is constrained on the unperturbed, no interacting, diene (DN) and dienophile (DP), obtained from their geometries at the transition state or on an IRC point, calculated at the M06-2X/6-31+G(d) level of theory.

$$\psi^0 = \begin{pmatrix} \psi_{DN}^0 & 0 \\ 0 & \psi_{DP}^0 \end{pmatrix} \quad (2)$$

ψ_{DN}^0 and ψ_{DP}^0 wavefunctions are orthogonal.

We also run electron dynamics on reactants geometries calculated by means of the LC-wPBE[7] long-range corrected functional, as implemented in Gaussian09:[2] the obtained bond order between fragments does not increase above 0.02 during the simulation, demonstrating that the applied local exchange-correlation functional (M06-2X) in the present study is suitable to describe the orbital overlap even on well separated reaction centers. The ground state Hamiltonian of the total interacting system, build up from its own eigenstates calculated at the same level of theory, is applied on the initial diabatic wavefunction as a constrained position-dependent perturbation, representing the electronic coupling interaction driving the electron dynamics. The coefficients of ψ(t) were updated at each time step, according to the implemented algorithm, and the total density matrix in time P(t) was calculated as the sum of the diene and dienophile density matrices, P_{DN}(t) and P_{DP}(t), respectively obtained as the product of their total molecular orbital coefficients:

$$P_{DN}(t) = \sum_{i=1}^{nocc(DN)} c_{v,i}^{*}(t)c_{i,v}(t);\;\; P_{DP}(t)\sum_{k=1}^{nocc(DP)} c_{v,k}^{*}(t)c_{k,v}(t) \qquad (3)$$

During the simulation, the total electron numbers is conserved, and no net charge is transferred between molecular fragments.

The time-dependent inter- and intra-molecular bond order between atoms A and B were calculated from the total density matrix P(t), according to the Wiberg definition [8-10]

$$BO_{AB}(t) = \sum_{\mu\epsilon A,v\epsilon B} P_{\mu v}(t)P_{v\mu}(t) \qquad (4)$$

The time-dependent atomic effective charge on atom A, for an atom centered basis set, was estimated by the Lödwin population analysis:

$$q_A(t) = Z_a - \sum_{i\epsilon A} P_{ii}(t) \qquad (5)$$

The orbital occupation numbers, determined by projecting the time-dependent density matrix onto the initial orbitals, were also calculated to track the process in terms of orbital symmetry:

$$n_k(t) = \psi_k^{*}(0)P(t)\psi_k(0) \qquad (6)$$

Table S1. Energies relative to reactants (kcal mol^{-1}), computed at the M06-2X/6-31+G(d) level, of the transition states corresponding to different approaches of 1-methoxy-butadiene and cyano-ethylene. Energy values include ZPE corrections. Approach **a** is the favored orientation discussed in the paper.

	**Relative orientation of the two substrate molecules**	**Energy**
**a**	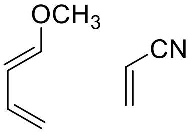	11.2
**b**	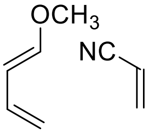	13.1
**c**	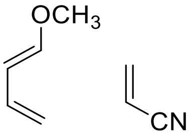	15.0
**d**	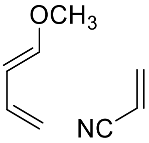	15.6
**e**	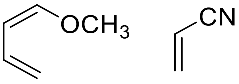	16.8
**f**	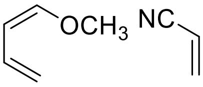	17.0
**g**	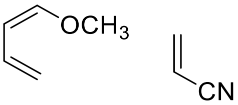	17.8
**h**	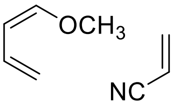	19.6

Table S2. Bond order (BO) values for the bonds of system **1**. Maximum, minimum and average BO's were calculated over 5 fs dynamics on TS_1. BO's for reactants (products) are obtained from DFT computations.

	C_1C_2	C_2C_3	C_5C_6	C_4C_5
**Rx_1 (Pd_1)**	2.11 (1.15)	1.23 (2.04)	2.23 (1.15)	0.0 (1.13)
**Maximum TS_1**	2.03	1.80	2.27	0.83
**Minimum TS_1**	1.31	1.14	1.21	0.0
**Average TS_1**	1.60	1.42	1.65	0.42

Table S3. Energies relative to reactants (kcal mol^{-1}) computed for transition state and along the IRC in the reactant direction for system **1** and **2**. Energy values include ZPE corrections.

	IP_1	IP_2	IP_3	IP_4	IP_5	Transition state
**System 1**	8.8	11.3	14.0	16.8	19.2	20.3
**System 2**	9.0	14.2	16.4	17.3	17.9	18.2

Table S4. Löwdin charges for system **1.** Maximum, minimum and average charges Q were calculated over the 5 fs dynamics.

	C_1 (C_4)	C_2 (C_3)	C_5 (C_6)
**Rx_1 (Pd_1)**	-0.42 (-0.41)	-0.27 (-0.28)	-0.44 (-0.42)
**Maximum TS_1**	-0.32	-0.03	-0.16
**Minimum TS_1**	-0.68	-0.51	-0.67
**Average TS_1**	-0.47	-0.28	-0.42

Table S5. Percentages of the dynamics computed for different ranges of Qs at the transition state TS_1 and for various points IP_n determined along the IRC in the reactant direction.

Charge range	TS_1	IP_1	IP_2	IP_3	IP_4	IP_5
C_4]-0.57 ; -0.37[C_5]-0.52 ; -0.32[	51%	69%	84%	91%	94%	96%
C_4]-0.57 ; -0.37[C_5 > -0.32	15%	8%	1%	0%	0%	0%
C_4]-0.57 ; -0.37[C_5 < -0.52	18%	9%	4%	1%	0%	0%

Comments to Table S5 . For 51% of dynamics the Q's on C_1 (C_4) range between -0.57 and -0.37 and the Q's on C5 (C6) range between -0.52 and -0.32. For 15% of dynamics C_5 (C_6) become more positive (Q > -0.32) and for 18% of dynamics C_5 (C_6) become more negative (Q < -0.52). These data emphasize the charge transfer occurring between the two fragments (in both directions) and that it is

necessary to invoke ~~also~~ the contribution of structures such as V, VI and VII to provide a more accurate description of the resonance hybrid corresponding to transition state.

Dynamics carried out along the IRC shows that, as the system approaches the reactants, the percentages of points corresponding to charges of C_5 (C_6) > -0.32 (increase of positive charge on ethene) and C_5 (C_6) < -0.52 (increase of negative charge) reach rapidly zero. This is consistent with the fact that a significant increase of positive or negative charges on C_5 (C_6) indicates an electron transfer between the two fragments, which disappears when they move away from each other.

Table S6. Bond order (BO) values for the bonds of system **2.** Maximum, minimum and average BOs were calculated over 5 fs dynamics. BO's for reactants (products) are obtained from DFT computations.

	**C_1C_2**	**C_2C_3**	**C_3C_4**	**C_5C_6**	**C_1C_6**	**C_4C_5**
**Rx$_2$<br>(Pd$_2$)**	1.89<br>(1.07)	1.21<br>(2.01)	2.09<br>(1.15)	2.10<br>(1.09)	0.0<br>(1.01)	0.0<br>(1.13)
**Maximum TS$_2$**	1.74	1.71	1.99	2.05	0.39	0.90
**Minimum TS$_2$**	1.22	1.18	1.24	1.18	0.00	0.00
**Average TS$_2$**	1.47	1.44	1.51	1.49	0.20	0.55

Table S7. Löwdin charges of system **2.** Maximum, minimum and average charges were calculated over the 5 fs of the dynamics.

	**C_1**	**C_2**	**C_3**	**C_4**	**C_6**	**C_5**
**Rx$_2$<br>(Pd$_2$)**	-0.08<br>(-0.08)	-0.29<br>(-0.25)	-0.25<br>(-0.24)	-0.44<br>(-0.40)	-0.23<br>(-0.24)	-0.34<br>(-0.38)
**Maximum TS$_2$**	0.08	-0.10	0.03	-0.24	-0.09	-0.23
**Minimum TS$_2$**	-0.24	-0.46	-0.51	-0.66	-0.49	-0.56
**Average TS$_2$**	-0.09	-0.28	-0.26	-0.44	-0.27	-0.41

Table S8. Mulliken charges for system **1**. Maximum, minimum and average charges were calculated over the 5 fs dynamics.

	**C_1 (C_4)**	**C_2 (C_3)**	**C_5 (C_6)**
**Rx$_1$<br>(Pd$_1$)**	-0.51<br>(-0.55)	-0.08<br>(-0.08)	-0.39<br>(-0.42)
**Maximum TS$_1$**	3.34	3.49	0.96
**Minimum TS$_1$**	-3.95	-4.03	-1.74
**Average TS$_1$**	-0.31	-0.31	-0.45

Comments to Table S8 . The Mulliken charges vary greatly and reach values of up to ±4 for the butadiene carbons. These values are unphysical and were not further considered for the analysis. Löwdin charges differ greatly from the Mulliken ones. The computation of the Löwdin charges implies the orthogonalization of the density matrix. The computation of the Mulliken charges leaves unchanged the density matrix: for this reason, the charges include inter-fragment contributions that should not be considered.

Table S9. Cartesian coordinates (Ångströms) for reactant, products, transition state and the various points along the IRC computed at the M06-2X/6-31+G(d) level for system **1.**

Butadiene

scf done: -155.9048688787

C	-1.279622	1.708235	0.192623
H	-1.025914	0.833845	-0.401952
H	-2.315981	1.818299	0.497739
C	-0.358423	2.621448	0.517002
H	-0.666400	3.509299	1.068606
C	1.069001	2.533667	0.164030
H	1.584600	3.479896	0.004027
C	1.756224	1.391684	0.055238
H	1.294865	0.428262	0.260552
H	2.804362	1.390706	-0.228041

Ethylene

scf done: -78.541590926

C	-0.691860	-0.854842	2.676542
H	-0.733978	-1.913143	2.920474
H	-1.559746	-0.430027	2.178143
C	0.368084	-0.107490	2.975315
H	0.405562	0.949037	2.722154
H	1.237254	-0.525531	3.476824

TS1

scf done = -234.421135364

C	-0.707000	1.385000	-0.015000
H	-1.773000	1.271000	0.161000
H	-0.285000	0.688000	-0.730000
C	-0.085000	2.582000	0.258000
H	-0.655000	3.372000	0.743000
C	1.318000	2.717000	0.192000
H	1.769000	3.606000	0.627000
C	2.128000	1.658000	-0.150000
H	3.208000	1.751000	-0.076000
C	0.237000	0.025000	1.531000
H	-0.255000	-0.830000	1.077000
H	-0.314000	0.516000	2.326000
C	1.610000	0.152000	1.461000
H	2.142000	0.738000	2.200000
H	2.199000	-0.603000	0.948000
H	1.780000	0.888000	-0.831000

IP1

scf done: -234.422564

C	-1.478035	0.026399	-0.601064
H	-2.541738	-0.094357	-0.415808
H	-1.035783	-0.702822	-1.271003
C	-0.846670	1.196062	-0.302088
H	-1.407571	1.984085	0.196263
C	0.570977	1.332629	-0.369633
H	1.016447	2.217636	0.080088

C	1.382626	0.302381	-0.737927
H	2.462512	0.388311	-0.654665
C	-0.502541	-1.389497	1.004259
H	-1.013514	-2.212317	0.511616
H	-1.071200	-0.864240	1.763663
C	0.850566	-1.265296	0.934300
H	1.390607	-0.640192	1.636859
H	1.448689	-1.985138	0.381989
H	1.026282	-0.503676	-1.369894

IP2

scf done: -234.426087

C	-1.496077	0.052539	-0.629550
H	-2.559045	-0.068577	-0.441780
H	-1.037474	-0.704887	-1.255112
C	-0.853399	1.196943	-0.303487
H	-1.407701	1.985548	0.201235
C	0.577281	1.334724	-0.371924
H	1.017017	2.219238	0.084351
C	1.392622	0.331135	-0.767929
H	2.472075	0.417241	-0.682167
C	-0.491504	-1.419827	1.035808
H	-1.015253	-2.214500	0.511681
H	-1.072203	-0.862075	1.762198
C	0.848396	-1.297154	0.966328
H	1.391087	-0.637854	1.634709
H	1.450611	-1.986821	0.381194
H	1.030700	-0.505837	-1.354157

IP3

scf done: -234.430454

C	-1.514285	0.077709	-0.657863
H	-2.578808	-0.036692	-0.475197
H	-1.044353	-0.703533	-1.243836
C	-0.858811	1.199130	-0.304834
H	-1.407859	1.990892	0.200820
C	0.581917	1.337847	-0.373923
H	1.016147	2.224467	0.083719
C	1.403010	0.358946	-0.797595
H	2.482167	0.452512	-0.717037
C	-0.482882	-1.450833	1.067495
H	-1.014715	-2.223705	0.519721
H	-1.071329	-0.865213	1.764950
C	0.848863	-1.328968	0.998159
H	1.391018	-0.641282	1.637539
H	1.452428	-1.996165	0.389190
H	1.038994	-0.503024	-1.344526

IP4

scf done: -234.434702

C	-1.531623	0.101898	-0.685155
H	-2.599401	0.000464	-0.514202
H	-1.056542	-0.697707	-1.241641
C	-0.862747	1.202923	-0.306838
H	-1.407367	1.999529	0.196261
C	0.584723	1.342243	-0.376309
H	1.013462	2.232732	0.079314

C	1.412878	0.385918	-0.826326
H	2.491545	0.493116	-0.757462
C	-0.476200	-1.482304	1.098955
H	-1.012394	-2.241774	0.536595
H	-1.069077	-0.876231	1.774972
C	0.851282	-1.360923	1.029716
H	1.391699	-0.652408	1.647599
H	1.455086	-2.014937	0.406619
H	1.050589	-0.494658	-1.344292

IP5

scf done: -234.438371

C	-1.547378	0.125340	-0.711039
H	-2.618960	0.041054	-0.555506
H	-1.072468	-0.687864	-1.248416
C	-0.865677	1.208408	-0.310180
H	-1.406212	2.009981	0.189688
C	0.586117	1.348136	-0.379837
H	1.009491	2.242724	0.073071
C	1.421430	0.411954	-0.853435
H	2.498993	0.536853	-0.799986
C	-0.470782	-1.514275	1.130095
H	-1.008542	-2.267891	0.561133
H	-1.066013	-0.894417	1.791550
C	0.855020	-1.393054	1.060901
H	1.393500	-0.670354	1.664046
H	1.458666	-2.041400	0.431501
H	1.064096	-0.481595	-1.353116

Table S10. Cartesian coordinates (Ångstroms) for reactant, products, transition state and the various points along the IRC computed at the M06-2X//6-31+G(d) level for system **2.**

Cyanoethylene

scf done: -170.759618

C	1.779343	-0.359253	2.106494
H	2.375533	0.528912	1.919474
C	1.383349	-0.698316	3.334917
H	0.787235	-1.587365	3.515156
H	1.651969	-0.086392	4.189280
C	1.441851	-1.147593	0.951621
N	1.177981	-1.771542	0.013024

1-Methoxy-butadiene

scf done: -270.386075

C	-1.328065	1.826089	0.052780
H	-2.371330	2.125643	0.037823
H	-1.117421	0.759620	0.033852
C	-0.345113	2.732543	0.114059
H	-0.609908	3.786163	0.201263
C	1.089681	2.430346	0.075342
H	1.772136	3.039703	0.663482
C	1.599300	1.448681	-0.677269
H	0.979233	0.853168	-1.351226

O	2.912708	1.117211	-0.638302
C	3.383142	0.413851	-1.772852
H	4.417085	0.138792	-1.564061
H	2.791826	-0.495312	-1.941326
H	3.345032	1.045386	-2.668277

TS2

scf done: -441.131252

C	-0.836563	1.293756	0.447337
H	-1.781012	1.088501	0.944877
H	-0.597267	0.592422	-0.348458
C	-0.403393	2.611911	0.371167
H	-0.919423	3.357145	0.973057
C	0.805291	2.999699	-0.227076
H	1.188935	4.004667	-0.072152
C	1.626227	2.104866	-0.872782
H	1.278138	1.150775	-1.262079
C	1.809205	0.475407	1.127020
H	2.575881	1.168619	1.451204
C	0.540213	0.449510	1.703388
H	0.364747	1.073841	2.572841
H	-0.005665	-0.490432	1.702623
O	2.832276	2.503027	-1.292974
C	3.659335	1.514594	-1.900103
H	4.156520	0.916171	-1.131635
H	3.069251	0.853486	-2.543236
H	4.398972	2.054194	-2.491233
C	2.206677	-0.579893	0.258076
N	2.492654	-1.412269	-0.502861

IP1

scf done: -441.132575

C	-2.365084	0.090924	0.555359
H	-3.301657	-0.121691	1.063676
H	-2.101491	-0.625562	-0.218765
C	-1.917080	1.394661	0.495323
H	-2.425993	2.140020	1.103370
C	-0.701140	1.784530	-0.104772
H	-0.316461	2.787450	0.060353
C	0.113897	0.898569	-0.756601
H	-0.227690	-0.064366	-1.130096
C	0.302402	-0.749087	1.264189
H	1.063563	-0.038046	1.563635
C	-0.951549	-0.777696	1.844888
H	-1.143702	-0.137524	2.698641
H	-1.522199	-1.702320	1.820782
O	1.324880	1.289578	-1.170681
C	2.149738	0.299201	-1.776441
H	2.646737	-0.299696	-1.008055
H	1.559268	-0.361380	-2.419724
H	2.890521	0.836778	-2.368097
C	0.697087	-1.797032	0.383117
N	0.983430	-2.627144	-0.379248

IP2

scf done: -441.135397

C	-2.384680	0.102555	0.539144

H	-3.315561	-0.114223	1.055242
H	-2.100598	-0.625733	-0.215106
C	-1.921257	1.394052	0.494833
H	-2.424432	2.139923	1.107121
C	-0.698512	1.785366	-0.106134
H	-0.312558	2.786136	0.068425
C	0.111499	0.907079	-0.764906
H	-0.225364	-0.062136	-1.126192
C	0.306850	-0.758839	1.277007
H	1.061650	-0.032195	1.553178
C	-0.935250	-0.789830	1.863184
H	-1.139952	-0.133828	2.700895
H	-1.524984	-1.700445	1.820746
O	1.327266	1.291710	-1.173054
C	2.149469	0.299004	-1.777502
H	2.645389	-0.300952	-1.009016
H	1.558090	-0.360577	-2.420993
H	2.891981	0.833825	-2.369490
C	0.697243	-1.799878	0.384297
N	0.983703	-2.626793	-0.380629

IP3

scf done: -441.138721

C	-2.404234	0.114064	0.522757
H	-3.330422	-0.106065	1.045355
H	-2.100394	-0.625928	-0.211677
C	-1.925508	1.393639	0.493906
H	-2.423558	2.140150	1.109772
C	-0.696061	1.786178	-0.107390
H	-0.309126	2.784981	0.075935
C	0.109346	0.915170	-0.772770
H	-0.223421	-0.059589	-1.123307
C	0.311567	-0.768470	1.289768
H	1.060161	-0.027588	1.544550
C	-0.919101	-0.802081	1.881729
H	-1.135809	-0.131022	2.703892
H	-1.527444	-1.698923	1.821125
O	1.329490	1.294095	-1.175664
C	2.149264	0.298947	-1.778534
H	2.644250	-0.301660	-1.009789
H	1.557166	-0.359895	-2.422117
H	2.893429	0.831164	-2.370887
C	0.697562	-1.802772	0.385616
N	0.983965	-2.626521	-0.381931

IP4

scf done: -441.141996

C	-2.443553	0.136540	0.490000
H	-3.364898	-0.085329	1.019704
H	-2.105598	-0.624166	-0.207614
C	-1.934253	1.394413	0.490625
H	-2.424393	2.143130	1.110487
C	-0.691641	1.788157	-0.108806
H	-0.302546	2.782998	0.091246
C	0.105951	0.930006	-0.787196
H	-0.221491	-0.052266	-1.122708
C	0.322509	-0.787596	1.314596
H	1.060231	-0.024296	1.533847

C	-0.888106	-0.826749	1.919938
H	-1.124185	-0.127519	2.712698
H	-1.528641	-1.698072	1.828028
O	1.333668	1.299600	-1.181719
C	2.148735	0.299177	-1.780976
H	2.640950	-0.302769	-1.011194
H	1.555012	-0.358110	-2.424687
H	2.896733	0.825610	-2.373859
C	0.698991	-1.809796	0.389261
N	0.984462	-2.626153	-0.385107

IP5

scf done: -441.144851

C	-2.535585	0.192163	0.410331
H	-3.472109	-0.007439	0.921791
H	-2.149113	-0.609468	-0.213687
C	-1.958449	1.406881	0.470755
H	-2.443480	2.168653	1.079186
C	-0.682515	1.791670	-0.104539
H	-0.286501	2.775933	0.131342
C	0.101188	0.958534	-0.814769
H	-0.226866	-0.026444	-1.146663
C	0.356963	-0.836826	1.373552
H	1.081561	-0.048421	1.546059
C	-0.817823	-0.887170	2.016362
H	-1.073764	-0.143442	2.761387
H	-1.510002	-1.711228	1.876198
O	1.339892	1.316475	-1.200861
C	2.145894	0.302910	-1.788970
H	2.627116	-0.302226	-1.014192
H	1.549088	-0.350479	-2.433960
H	2.905011	0.815355	-2.380113
C	0.707004	-1.833593	0.404725
N	0.984517	-2.625574	-0.395507

Pd2

scf done: -441.224486

C	-0.547167	1.317343	0.909858
H	-0.898167	1.697816	1.875739
H	-1.268724	0.546262	0.603031
C	-0.548318	2.432306	-0.100251
H	-1.406229	3.101923	-0.100945
C	0.434229	2.636307	-0.979977
H	0.396542	3.479942	-1.664597
C	1.646582	1.753007	-1.096568
H	1.819319	1.516201	-2.158715
C	1.467900	0.429898	-0.312318
H	2.451693	-0.036728	-0.188040
C	0.844952	0.710004	1.064548
H	1.507363	1.419917	1.572731
H	0.809740	-0.207949	1.658629
O	2.762689	2.466414	-0.595655
C	3.998215	2.062180	-1.145658
H	4.224993	1.009573	-0.925885
H	4.008909	2.206356	-2.234630
H	4.766754	2.688936	-0.690885
C	0.638865	-0.510265	-1.082116

N -0.023870 -1.241174 -1.685757

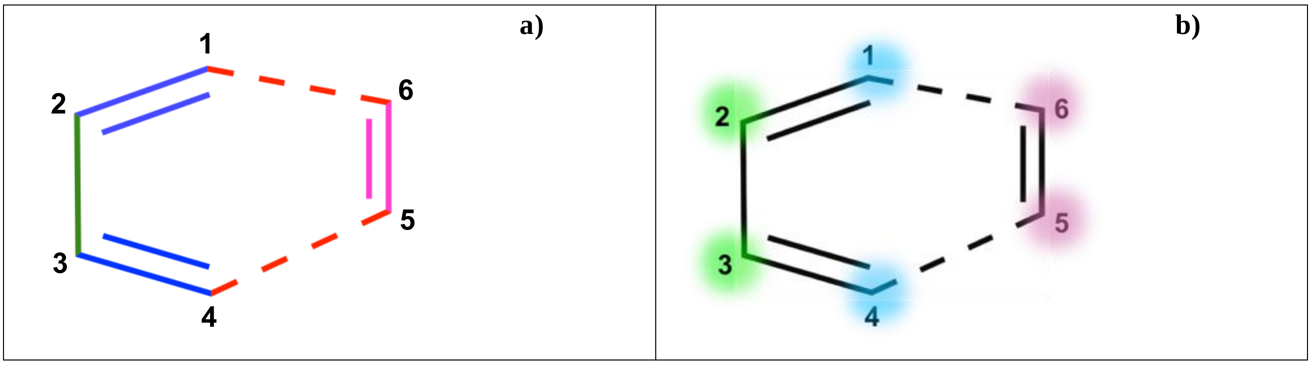

Scheme S1. Color code for **a)** bond orders, **b)** atomic charges of system **1** used in Figure S1.

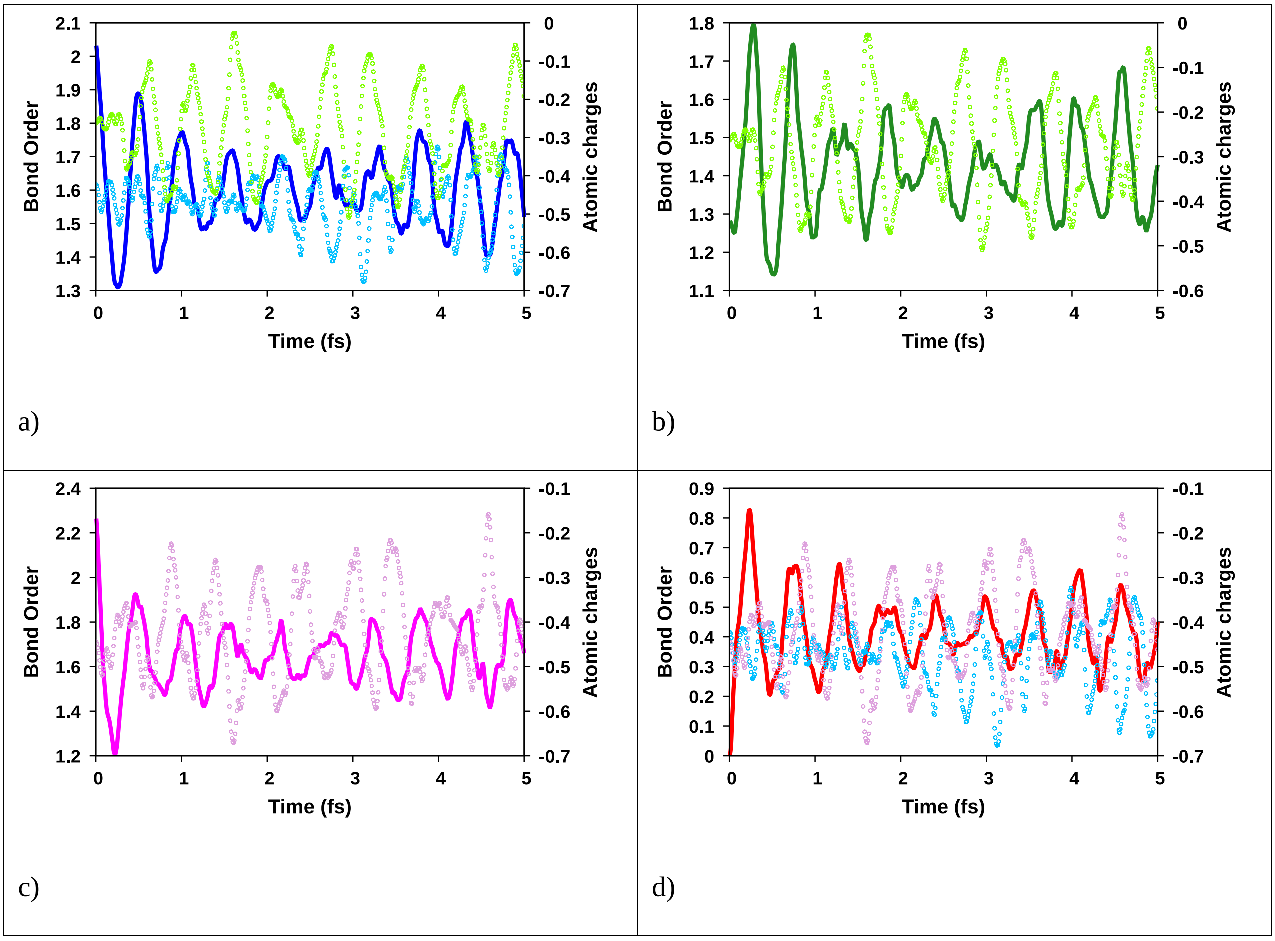

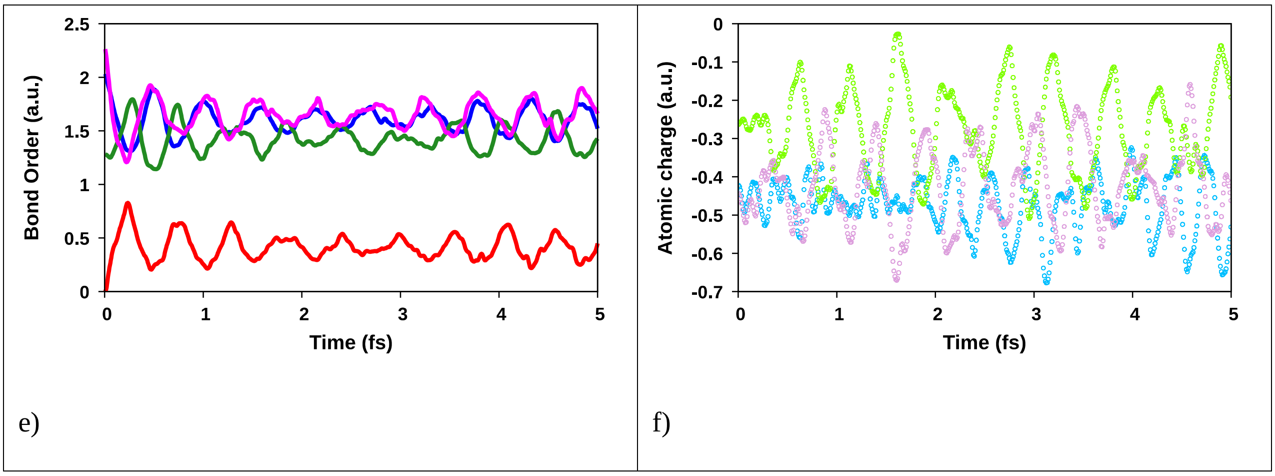

Figure S1. System 1. Left axis: bond order (solid lines); Right axis: Löwdin atomic charges of the atoms involved in the bond (dotted lines); a) C_1-C_2 bond order (blue); C_1 (light blue) and C_2 (light green) charges; b) C_2-C_3 bond order (dark green); C_2/C_3 (light green) charges; c) C_5-C_6 bond order (magenta); C_5/C_6 (plum) charges; d) C_1-C_6 bond order (red); C_1 (light blue) and C_6 (plum) charges; e) comparison of the bond orders, color coding as above; f) comparison of the charges, color coding as above.

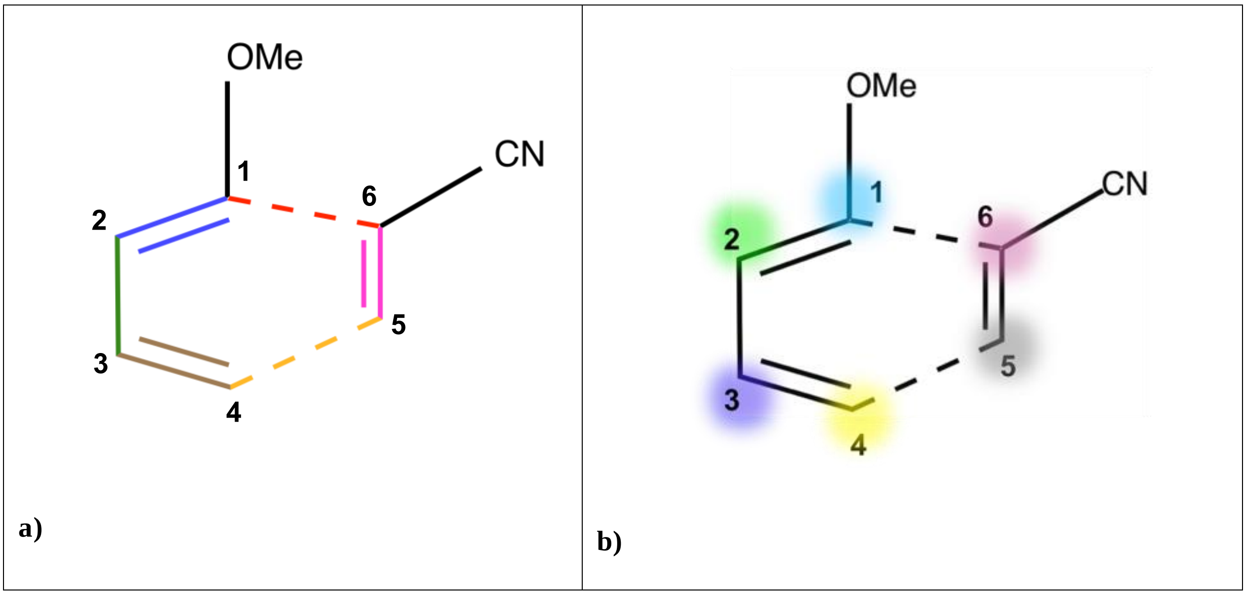

Scheme S2. Color code for **a)** bond orders, **b)** atomic charges of system **2** used in Figure S2.

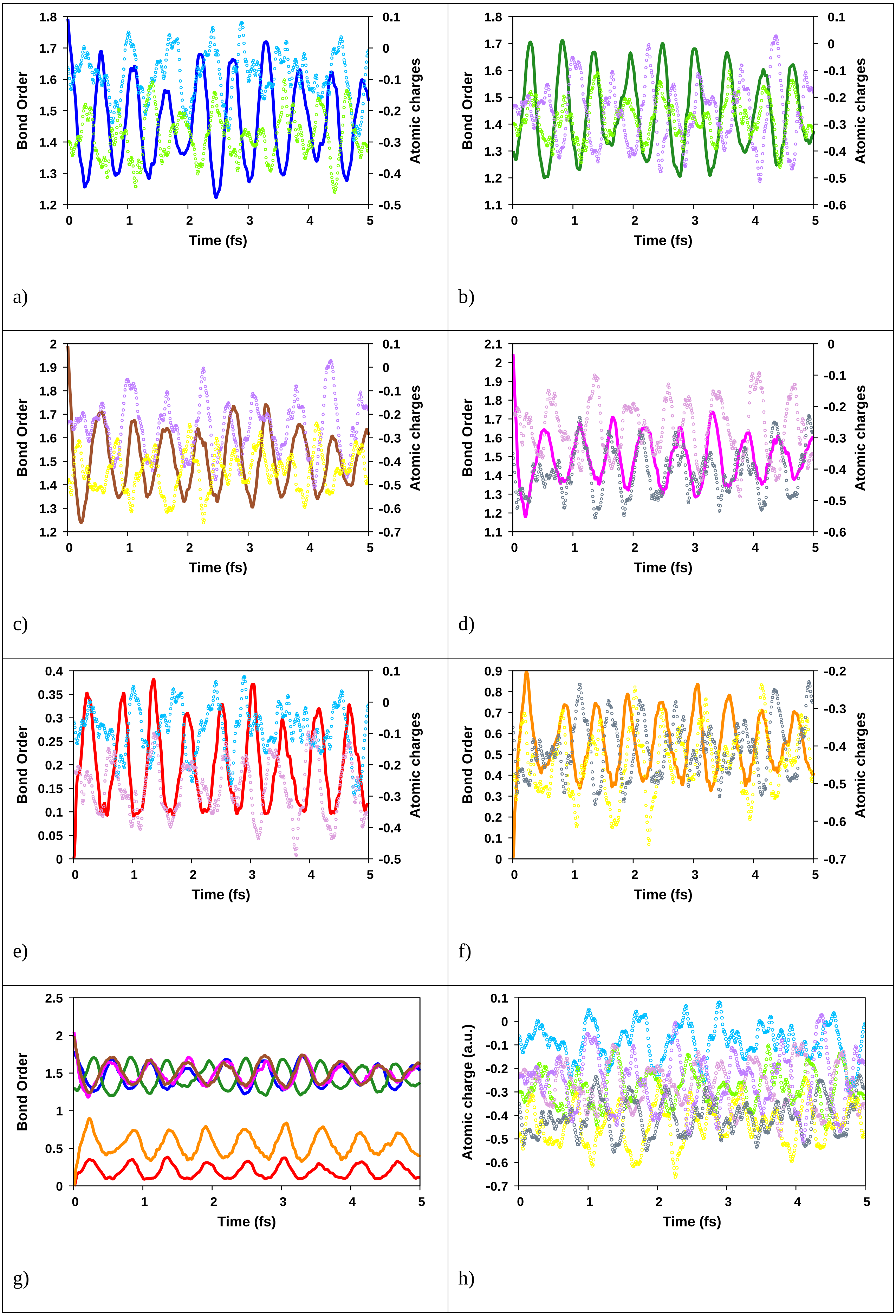
Bond Order
Atomic charges
Time (fs)
a)
b)
c)
d)
e)
f)
g)
h)
Atomic charge (a.u.)

Figure S2. System 2. Left axis: bond order (solid lines); Right axis: Lowdin atomic charges of the atoms involved in the bond (dotted lines); a) C_1-C_2 bond order (blue); C_1 (light blue) and C_2 (light green) charges; b) C_2-C_3 bond order (dark green); C_2 (light green) and C_3 (violet) charges; c) C_3-C_4 bond order (brown); C_3 (violet) and C_4 (yellow) charges; d) C_5-C_6 bond order (magenta); C_6 (plum) and C_5 (gray) charges; e) C_1-C_6 bond order (red); C_1 (light blue) and C_6 (plum) charges; f) C_4-C_5 bond order (orange); C_4 (yellow) and C_6 (gray) charges; g) comparison of the bond orders, color coding as above; h) comparison of the charges, color coding as above.

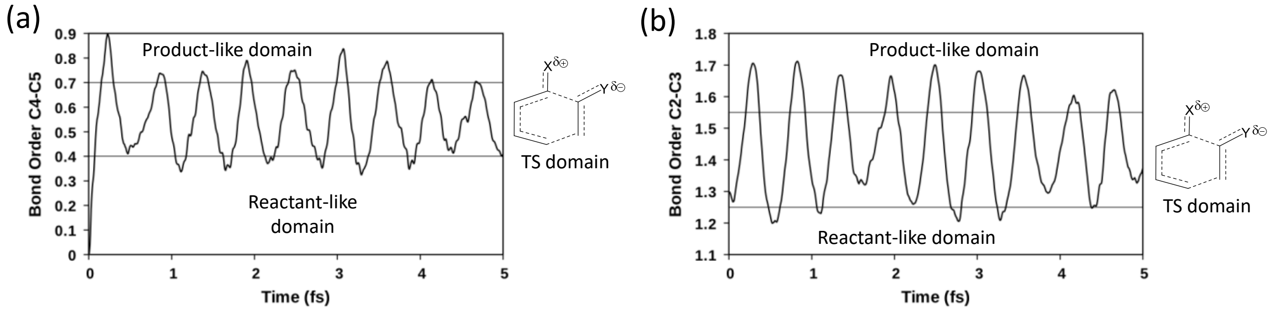

Figure S3. Time-dependency of (a) C_4-C_5 (C_1-C_6) BO and (b) C_2-C_3 BO. The transition state (benzene-like) domain corresponds to the central horizontal zone.

References

1. C. O. N. Spectus, Density Functionals with Broad Applicability in Chemistry. **41,** 157–167 (2008).
2. Gaussian 09, Frisch, M. J.; Trucks, G. W.; Schlegel, H. B.; Scuseria, G. E.; Robb, M. A.; Cheeseman, J. R.; Scalmani, G.; Barone, V.; Mennucci, B.; Petersson, G. A. *et al.* Gaussian, Inc., Wallingford CT, 2009.
3. Nziko, V. D. P. N. & Scheiner, S. Catalysis of the Aza-Diels-Alder Reaction by Hydrogen and Halogen Bonds. *J. Org. Chem.* **81,** 2589–2597 (2016).
4. Pieniazek, S. N., Clemente, F. R. & Houk, K. N. Sources of error in DFT computations of C-C bond formation thermochemistries: π→σ transformations and error cancellation by DFT methods. *Angew. Chemie - Int. Ed.* **47,** 7746–7749 (2008).
5. Pieniazek, S. N. & Houk, K. N. The origin of the halogen effect on reactivity and reversibility of Diels-Alder cycloadditions involving furan. *Angew. Chemie - Int. Ed.* **45,** 1442–1445 (2006).
6. Paton, R. S. *et al.* Origins of Stereoselectivity in the trans Diels-Alder Paradigm. *J. Am. Chem. Soc.* **132,** 9335–9340 (2010)
7. Henderson, T. M., Izmaylov, A. F., Scalmani, G. & Scuseria, G. E. Can short-range hybrids describe long-range-dependent properties? *J. Chem. Phys.* **131,** 0–9 (2009).
8. Bridgeman, A. J., Cavigliasso, G., Ireland, L. R. & Rothery, J. The Mayer bond order as a tool in inorganic chemistry†. *J. Chem. Soc. Dalt. Trans.* 2095–2108 (2001). doi:10.1039/b102094n
9. Kalinowski, J. A., Lesyng, B., Thompson, J. D., Cramer, C. J. & Truhlar, D. G. Method. 2545–2549 (2004).
10. Bochiccio, R.C., Reale, H.F. On the nature of crystalline bonding : extension of statistical population analysis to two- and three- dimensional crystalline systems. *J. of Physics B: At. Mol. Opt. Phys.* **26**, 4871-4883 (1993).
